# Interest communities and flow roles in directed networks: the Twitter network of the UK riots

**DOI:** 10.1098/rsif.2014.0940

**Published:** 2014-12-06

**Authors:** Mariano Beguerisse-Díaz, Guillermo Garduño-Hernández, Borislav Vangelov, Sophia N. Yaliraki, Mauricio Barahona

**Affiliations:** 1 Department of Mathematics, Imperial College London, London SW7 2AZ, UK; 2 Department of Chemistry, Imperial College London, London SW7 2AZ, UK; 3 Sinnia, Mexico City, Mexico

**Keywords:** community detection, flow roles, directed networks, Twitter, UK riots, graph theory and stochastic processes

## Abstract

Directionality is a crucial ingredient in many complex networks in which information, energy or influence are transmitted. In such directed networks, analysing flows (and not only the strength of connections) is crucial to reveal important features of the network that might go undetected if the orientation of connections is ignored. We showcase here a flow-based approach for community detection through the study of the network of the most influential Twitter users during the 2011 riots in England. Firstly, we use directed Markov Stability to extract descriptions of the network at different levels of coarseness in terms of interest communities, i.e. groups of nodes within which flows of information are contained and reinforced. Such interest communities reveal user groupings according to location, profession, employer and topic. The study of flows also allows us to generate an interest distance, which affords a personalized view of the attention in the network as viewed from the vantage point of any given user. Secondly, we analyse the profiles of incoming and outgoing long-range flows with a combined approach of role-based similarity and the novel relaxed minimum spanning tree algorithm to reveal that the users in the network can be classified into five roles. These flow roles go beyond the standard leader/follower dichotomy and differ from classifications based on regular/structural equivalence. We then show that the interest communities fall into distinct informational organigrams characterized by a different mix of user roles reflecting the quality of dialogue within them. Our generic framework can be used to provide insight into how flows are generated, distributed, preserved and consumed in directed networks.

## 1. Introduction

The increasing availability of large-scale relational datasets in a variety of fields has led to the widespread analysis of complex networks. In particular, the current interest in quantitative social sciences has been fuelled by the importance of social networks and by the wealth of socio-economic datasets widely available today [1–9]. Due to the sheer complexity of these networks, it has become crucial to develop tools for network analysis that can increase our insight into such data. A key direction in this area is that of *community detection*, which aims at extracting a simplified, yet meaningful, coarse-grained representation of a complex network in terms of ‘communities’ of nodes at different levels of resolution [10].

A common characteristic of many social, engineering and biological networks is the importance of directionality. Clearly, it is not the same to ‘follow’ a widely known personality in Twitter as to be followed by one. Directionality is also key in food webs [11], brain networks [12], economics datasets [13], protein interaction networks [13] and trade networks [14], to name but a few. Failure to consider directionality when present in the data, as is commonly done in numerous network analyses, entails ignoring the true nature of the asymmetric relationships and information propagation. From a methodological perspective, however, the analysis of directed networks presents unique challenges that put them beyond standard methodologies. In particular, it is difficult to extend the structural notion of community (i.e. a group of nodes with strong connectivity within and with weaker connectivity to the outside) to the case of directed networks.

Here we show how the analysis of flow patterns on a network can be integrated to provide a framework for community [15,16] and role [17] detection. This framework is naturally applicable to directed networks where flow is an intrinsic feature of the system they represent. Our analysis is able to reveal a layered view of the data from four complementary perspectives: interest communities of nodes at different levels of resolution; a personalized view of interest in the network from any vantage point; the identification of user roles in the network based on directed flows; and a classification of the interest communities into distinctive information organigrams. Our framework is applicable to generic directed networks, but we showcase our approach through the analysis of the Twitter network of influential Twitter users during the 2011 riots in England, compiled from the list published by the British newspaper *The Guardian*.

### 1.1. The directed network of influential Twitter users during the UK riots

The riots of 6–10 August 2011 in England were followed by an intense public debate about the role and influence of social media during the unrest. Politicians, journalists, pundits and bloggers alike weighed in on the issue, but few arguments were based on data [18]. A few months after the riots, *The Guardian* made available to the public a list of the 1000 ‘most influential’ (i.e. the most *re-tweeted*) Twitter users during the riots [19]. The list compiled by *The Guardian* comprised a diverse set of Twitter users, including newspapers, broadcasting services, news agencies, as well as individual accounts of journalists, politicians, entertainers, global and local activists, and members of the public.

To enable a quantitative analysis of *The Guardian's* list, we mined Twitter in February 2012 and recovered the *directed* network of followers within the list (see the electronic supplementary material). Henceforth we study the largest connected component of this network consisting of *N* = 914 nodes (Twitter users). The remaining 86 users were either disconnected (i.e. they did not follow nor were followed by anyone on the list) or their accounts had since been deleted. In our network, an edge indicates that the source node is subscribed to the *tweets* of the target node, i.e. the direction of the edge indicates the declared interest, whereas information and content travel in the opposite direction (electronic supplementary material, figure S1).

## 2. Results

### 2.1. Flow-based ‘interest communities’: a view of the network at different resolutions

**Figure 1.**
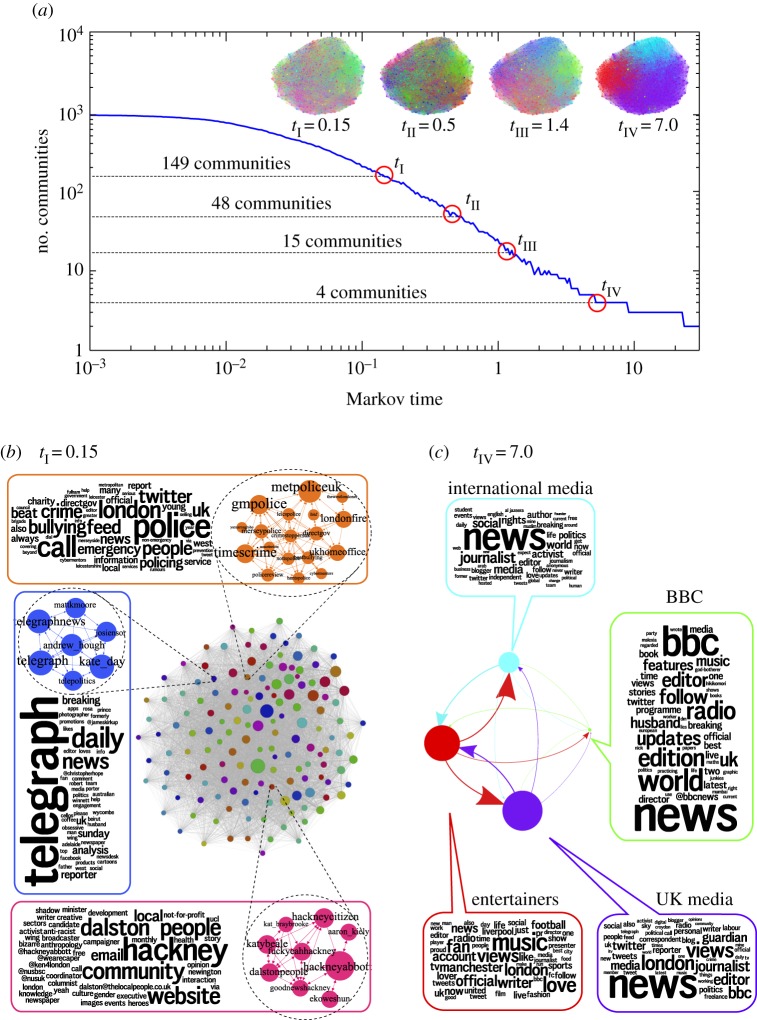
Interest communities at all scales as detected by Markov Stability. (*a*) The number of communities at each Markov time (*t*). The inset shows the network with nodes and edges coloured according to their community at four illustrative Markov times. Two of these partitions at different resolutions are shown in more detail. (*b*) At relatively short Markov times (*t*_I_ = 0.15), we find 149 communities (coarse-grained network view in the centre). Three examples of communities in this partition are ‘police and crime reporting’ (top), ‘Hackney’ (bottom), ‘the *Daily Telegraph*’ (left) shown with their members and their self-description word clouds. (*c*) At longer Markov times (*t*_IV_ = 7) we find four communities (coarse-grained view in the centre): three large communities broadly corresponding to ‘UK’ (bottom-right), ‘international’ (top), ‘celebrities/entertainment’ (bottom-left) and a small one corresponding to the ‘BBC’ (right). A detailed view of the partitions can be found in the electronic supplementary material.

To gain a structured view of the communities in the network at different levels of resolution, we use Markov Stability community detection [15,20] which has been extended to deal with directed networks (see Methods, electronic supplementary material and [16]). A key advantage of Markov Stability is that it is based on a quantitative criterion that relies on flow propagation and containment, and thus identifies *flow communities*. The communities so found correspond to ‘interest communities’, insomuch as information, interest and influence are propagated, retained and reinforced within them following the edges. If edge directionality is ignored, the community structure is blurred and the analysis severely hindered, as shown below. A second advantage of our method is that the network is scanned for structure at all scales, and flow communities are found to be relevant at different levels of resolution. Figure 1*a* illustrates how, as the network is swept by a continuous-time diffusion process, the method goes from detecting many small, granular communities (at short Markov times) to fewer and coarser communities (at longer Markov times). As a visual aid to interpret the theme of the communities, we create ‘word clouds’ from the most frequently used words in the Twitter self-biographies of the users in each community. It is important to remark that the biographies are not used in the network analysis, i.e. the word clouds serve as an independent, *a posteriori* annotation or ‘self-description’ of the communities found (see the electronic supplementary material).

An example of a highly granular partition (149 communities) at short Markov times is shown in figure 1*b* (electronic supplementary material, figures S3 and S4). At this resolution, some communities are defined by the geographical origin of the Twitter accounts (e.g. Midlands, Manchester, Liverpool, even Croydon and Hackney within London); others are determined by employer or institution (e.g. media such as *The Independent*, ITV, Channel 4 or the *Daily Telegraph*); while others correspond to interest groups (e.g. a community grouping together police forces and fire departments of riot areas with crime reporters and civil organizations highlights the police's use of Twitter during the riots [21]).

As the Markov time increases, we find coarser partitions with larger communities. At *t* = 0.5, we find 48 communities, including a football/sports community (clubs, athletes, sports journalists and supporters), a politics/Labour community and a relatively small BBC community (electronic supplementary material, figure S5). At a longer Markov time (*t* = 1.3), we find a partition into 15 communities, including the BBC community, a Sky community, a community of *Guardian* journalists, a community of international and alternative media/journalists/activists (including Wikileaks, Al Jazeera and Anonymous-related accounts), among other topical communities (§2.5).

At even longer Markov times, we show in figure 1*c* a coarse partition with four communities corresponding broadly to UK media/activism, international media/activism, entertainment/sports and the BBC, which remains as a distinct community across a large span of Markov times. We provide a spreadsheet in the electronic supplementary material with all partitions of the network at all Markov times so that interested parties can explore the all-scale structure of interest communities in the network. Furthermore, we have carried out a similar analysis using the well-known information-theoretic Infomap community detection algorithm [22,23], which in this case leads to an overpartitioned description with non-optimal compression (i.e. a large compression gap) *and* unbalanced partitions (see the electronic supplementary material for a discussion) [20,24].

### 2.2. The importance of directionality in detecting interest communities

In systems that are represented as directed networks, such as Twitter, the directionality of the edges is central to their function. The full consideration of edge directionality, which is naturally incorporated in our analysis, is crucial for the community structure detected. To illustrate this phenomenon, we compare the communities found in the original, directed Twitter network with those obtained when edge orientation is ignored. We have analysed both versions of the network (directed and undirected) using the extended Markov Stability method which can deal with both types of networks. See the electronic supplementary material, figure S6, for a discussion of the differences in community structure between the directed and undirected versions of this Twitter network. Importantly, relevant communities can go undetected if one uses standard approaches for community detection based on undirected structural notions (typically density of connections [24]).

**Figure 2.**
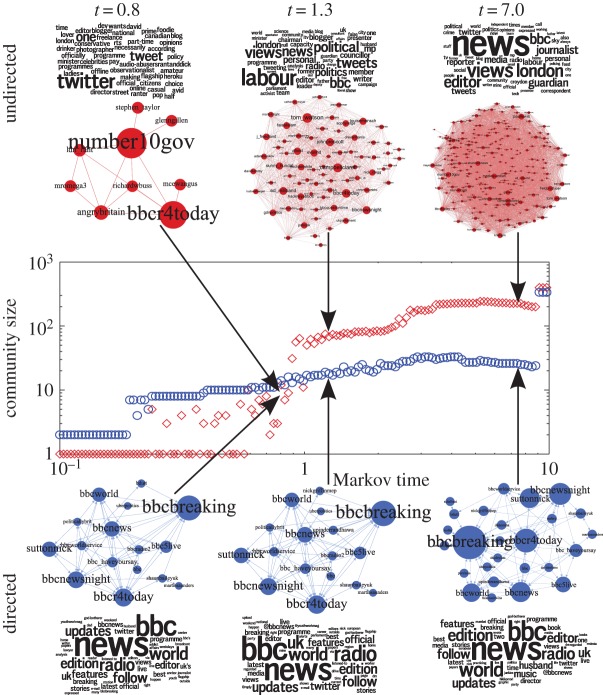
Communities containing the account of BBC Radio 4's *Today* programme (bbcr4today) in the undirected (top, diamonds) and directed (bottom, circles) versions of the network at Markov times *t* = 0.86, *t* = 1.3 and *t* = 7.0, along with their word clouds. In the middle we show the size of the communities of the *Today* programme in both versions of the network for Markov times between 10^−1^ and 10^1^. (Online version in colour.)

As stated above, the BBC is an example of a flow community that stands out in its persistency. In figure 2, we show how the community of BBC's *Today* programme (a morning news broadcast with a broad audience) remains consistently grouped across many levels of resolution in the analysis of the directed network: from an early Markov time, BBC-related accounts are grouped together and remain so all the way up to the top levels of resolution, with consistent word clouds throughout. This phenomenon depends strongly on the directionality of the flows: the nodes in the BBC community are among the most important in the network (high in-degree and PageRank), attracting flow (attention) from elsewhere in the network and retaining it for long periods of Markov time. In a symmetrized network, such communities can go undetected, as shown in figure 2, where the corresponding undirected community of the BBC's *Today* programme is quickly blurred across Markov times and gets mixed with a variety of users with little in common, consisting mainly of politicians from the Labour Party and journalists.

**Figure 3.**
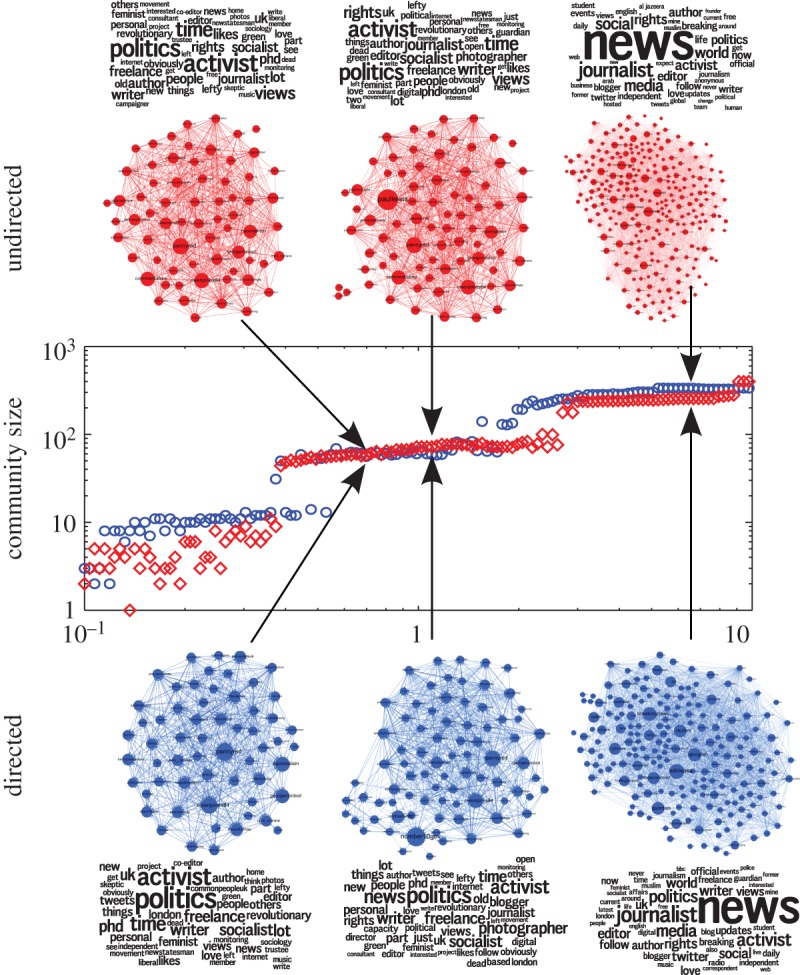
Directed and undirected communities containing the account of George Monbiot (georgemonbiot) obtained from the undirected (top, diamonds) and directed (bottom, circles) networks at Markov times *t* = 0.8603, *t* = 1.3049 and *t* = 7.0, along with their word clouds. Compare these results with those obtained in figure 2 for BBC Radio 4's *Today* programme. (Online version in colour.)

Interestingly, this drastic difference between directed and undirected communities is not observed for all communities in the network. There are communities, such as the one including *Guardian* columnist George Monbiot, which behave in an essentially similar manner in both cases across levels of resolution (figure 3). This difference between communities that are sensitive or insensitive to directionality persists across time scales, signalling the fact that some groupings (such as the BBC community) are fundamentally based on retention of directed flows, while others (such as the Monbiot community) follow from a balanced flow and, thus, can be captured by standard undirected measures. We note that the directed Markov Stability method is able to detect both types of communities simultaneously.

### 2.3. Interest distance between nodes: the view of the network from a vantage point

**Figure 4.**
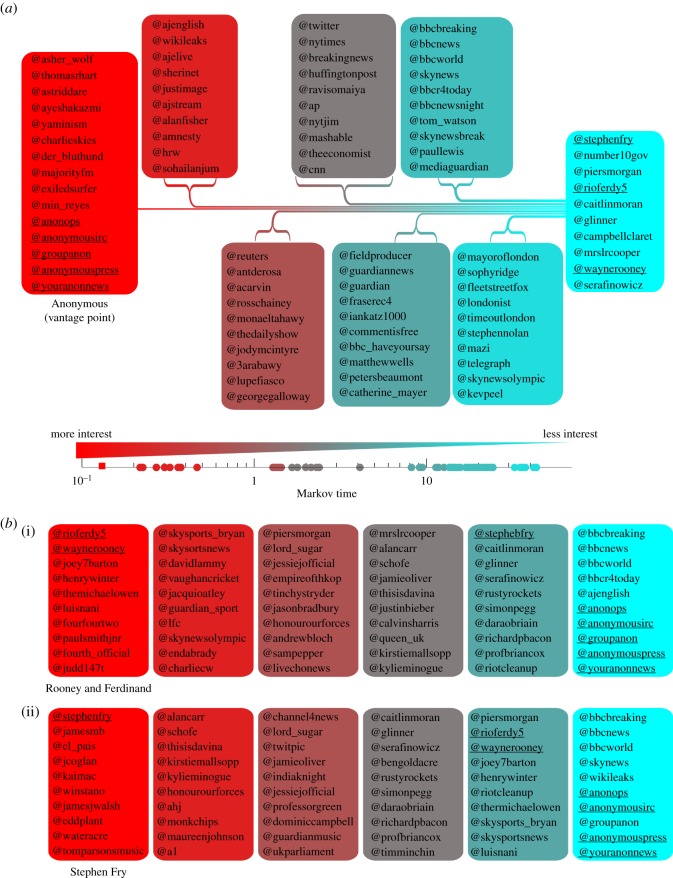
(*a*) Personalized view of the network from the vantage point of ‘Anonymous’ based on interest distance. The interest distance (gradient from red to blue, or dark to light in black and white) is defined as the earliest Markov time at which a node belongs in the same interest community as ‘Anonymous’. The number of users in the interest community of ‘Anonymous’ (represented by the width of the line) grows as the Markov time increases, as users join the community at different times. We show the top 10 users (according to PageRank) of every batch that joins the Anonymous community. (*b*) The reverse personalized views from two vantage points that are of least interest to ‘Anonymous’: (i) from the vantage point corresponding to Wayne Rooney and several footballers and (ii) from the vantage point of actor Stephen Fry. (Online version in colour.)

As the Markov diffusion explores the network, it can provide us with information of how interesting the members of the network are to a given node or group of nodes (denoted the ‘vantage point’). Using our flow-based communities, we establish the *interest distance* from the vantage point to any node in the network as the earliest Markov time at which the node belongs to the same community as the vantage point (i.e. we compute how ‘near’ they are in an ultrametric space [25]). In figure 4*a*, we show the computed interest distance from the vantage point of the Anonymous community (from *t* = 0.15 onwards). Consistent with other studies [26,27], the closest nodes to Anonymous include Wikileaks, Human Rights Watch, Al Jazeera and Amnesty International, followed by a mix of activists and writers, mainstream international media and the UK media. Of least interest to Anonymous are celebrities, UK politicians and footballers.

Unsurprisingly, the picture is starkly different from the vantage point of the nodes that are of least interest to Anonymous. Figure 4*b* shows the interest distance from the vantage point of footballer Wayne Rooney (of little interest to Anonymous), whose neighbourhood of interest is dominated by football, sports and TV celebrities, with news and activists as distant interests. The computed interest distance is able to capture the nuanced information provided by all the directed paths in the network. This is shown by the fact that Stephen Fry (English actor, TV personality and writer) is distant from *both* Wayne Rooney and Anonymous (figure 4*b*), while Rio Ferdinand (Rooney's ex-teammate at Manchester United) is always close to Rooney. These examples highlight the sensitivity of our Markov exploration and how the use of vantage points can be used to provide personalized information about the system.

### 2.4. Finding flow-based roles of nodes in directed networks

A flow-based analysis of directed networks also provides a different angle for the classification of nodes according to their role in generating and disseminating information. Conceptually, it is clear that an account with millions of followers, such as BBC News, acts as a source of information (i.e. a reference) while a personal account with only a handful of followers yet with subscriptions to media outlets acts mostly as a sink of information (i.e. a listener). To go beyond this source/sink dichotomy, or the traditional leader/follower and hub/authority [28] categories, we use here the full structure of flows in the network to develop a quantitative methodology that reveals ‘flow roles’ in the network without imposing the number of roles *a priori*. Our algorithm starts by building the *role-based similarity* (RBS) matrix (see Methods) [17,29]. A feature vector for each node *i* is constructed from the scaled pattern of incoming and outgoing paths of *all lengths* and the pairwise cosine similarities (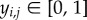) between all such vectors (see Methods) are stored in the *N* × *N* similarity matrix *Y*. Nodes with similar profiles of incoming and outgoing flows of all lengths are classified as having similar *flow roles* in the network (i.e. when *y_i,j_* is close to 1). The extreme cases correspond to the standard ‘sources’ and ‘sinks’, but an assortment of nuanced roles spanning these two extremes emerges in our results. This analysis provides a complementary use of flows to infer different properties of nodes: instead of grouping nodes according to flow persistence (as in the detection of interest communities described above), RBS provides a grouping of nodes according to their function in information propagation.

**Figure 5.**
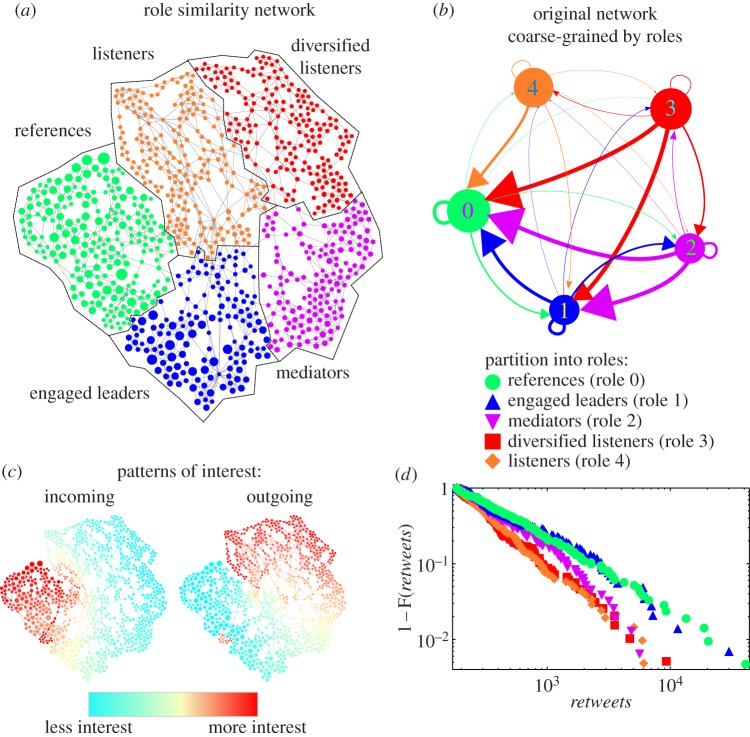
Flow-based roles in the Twitter network. (*a*) Role similarity graph obtained from the path similarity matrix using the RMST algorithm. The size of the nodes is proportional to the in-degree in the Twitter network. Nodes with similar profiles of in- and out-paths of all lengths in the original Twitter network are close in this role similarity graph. The role similarity graph is found to contain five robust clusters, corresponding to flow roles (see Methods and electronic supplementary material). (*b*) The original Twitter network coarse-grained according to roles, with arrows proportional to users in one role class who follow users in another role class. (*c*) Pattern of incoming and outgoing interest at all path lengths: (left) nodes in red (dark) receive the most attention with higher numbers of incoming paths, while nodes in blue (light) receive the least amount of attention; (right) nodes in red pay the most attention with higher numbers of outgoing paths, while nodes in blue pay the least amount of attention with few outgoing paths. (*d*) Cumulative distribution of retweets for each of the five roles: highly retweeted nodes are heavily present in the references and engaged leader categories (longer tails) and mostly absent in both listener categories. The mediator category lies in between.

We have extended the RBS method by using the relaxed minimum spanning tree (RMST) algorithm to extract a *role similarity graph* from the matrix *Y* (figure 5*a*). This novel algorithm creates a new graph by emphasizing strong similarities between nodes and downplaying weaker, redundant similarities based on local continuity and global geometric properties of the data similarity *Y* (see Methods). Note that in this RMST–RBS role similarity graph (which is generated from the Twitter graph but is distinct from it), nodes with similar connectivity patterns lie close to each other regardless of how close they are (in a geodesic way) in the original network. We then apply graph-theoretical community detection algorithms (such as Markov Stability) to the RMST–RBS graph and, in doing so, we reveal groups of nodes (the communities in the role similarity graph) with similar in- and out-flow patterns corresponding to *flow-based roles*. The number of communities on the role similarity graph corresponds to the number of roles in the network. Note that this procedure does not impose an *a priori* number of roles to be detected (see the electronic supplementary material).

Our RMST–RBS analysis finds that there are five flow-based roles for the nodes in this Twitter network. Examination of their incoming and outgoing flow patterns reveals that some of the groups identified correspond to traditional roles such as *listeners* (‘followers’) or *references* (‘leaders’) but also distinguishes between different types of leaders, followers and intermediate roles (figure 5*a,b*). The description of the five flow role categories we obtained is as follows.

*References*. Typically, institutional accounts, important sources of content or well-known personalities with many followers who follow few accounts, e.g. BBC Breaking News, Al Jazeera, Stephen Fry or *The New York Times*.
*Engaged leaders*. Accounts with large numbers of followers who, unlike references, also follow other users. This category includes institutional and personal accounts often meant to interact with the public, e.g. Sky News, the British Prime Minister's office, Tom Watson (a British MP) or Paul Lewis (*Guardian* editor).
*Mediators*. Users who interact with the two leader categories (i.e. they follow and are followed by high-profile accounts), as well as with nodes in the listener categories below. Many such accounts belong to journalists and reporters. Examples of mediators include Ross Chainey (Reuters employee), BBC-have-your-say and the London Fire Brigade.
*Diversified listeners*. Accounts with few followers that follow many nodes from all categories, suggesting diversity in their interests and sources of information.
*Listeners*. Accounts with few followers (within this network, not necessarily over the whole of Twitter) who follow mostly Reference nodes. Within this particular network, they can be considered as passive recipients of mainstream content.

In the spreadsheet in the electronic supplementary material, we give the roles of all nodes in the network. We remark that this classification of nodes into roles is pertinent *only* in the context of the network within the list compiled by *The Guardian*; it is possible that the role of certain users will be different if considered embedded in the wider Twitter network, since the pattern of paths of different lengths attached to each node is likely to change.

Figure 5*c* illustrates the mathematical basis for the classification of nodes into roles by our RMST–RBS algorithm: the patterns of incoming and outgoing flow at all path lengths are combined to reveal the different flow roles. Because RMST–RBS takes into account the whole spectrum of short to long paths (from length 1 to *K*_max_ = 133 in this case, and everything in between) our classification goes beyond similarity scores that only use single features, such as in- and out-degrees of the nodes (which appear as the paths of length 1 in columns 1 and *K*_max_ + 1 of the matrix *X*(*α*) in equation (A 5)) or eigencentrality-type stationary flow metrics (columns *K*_max_ and 2*K*_max_). Therefore, our method obtains information which is not apparent if we just rank the nodes according to in-/out-degree or centrality and then split them into groups. For example, ranking the nodes according to PageRank is not enough to distinguish the ‘Reference’ and ‘Engaged leader’ categories, or to separate ‘Mediator’ from ‘Engaged leader’ or ‘Diversified listener’ (see the electronic supplementary material, figure S8 and spreadsheet). To confirm the relevance of our findings, we examine the cumulative distribution of retweets attained by each node class (figure 5*d*), where we see a clear separation between the leader (reference and engaged leader nodes) and follower (diversified listeners and listeners) categories, with the mediators lying in between both groups. It is important to remark that the retweet data in figure 5*d* are not part of our role detection and are only used *a posteriori* to inform our understanding of the flow roles obtained (see also the electronic supplementary material, figure S8).

The flow roles we find here are conceptually and practically different from those obtained using well-established theories in social network analysis, e.g. structural equivalence (SE) [30] and regular equivalence (RE) [31–34]. SE bases node similarity on overlapping sets of neighbours (i.e. two nodes are similar if many of their neighbours are the same), whereas RE-based methods rely on node colorations and neighbourhoods (i.e. two nodes have the same role if the colours of their neighbours are the same, regardless of the number of common neighbours). Hence SE and RE are essentially short-path methods and not suitable for networks like the one studied here where the full structure of flow is essential (see the electronic supplementary material for a detailed description of RE and SE roles and their lack of information content in this network). Furthermore, RE methods are not robust to small random perturbations in network connectivity due to their combinatorial nature.

### 2.5. Interest communities and their distinct mix of roles

**Figure 6.**
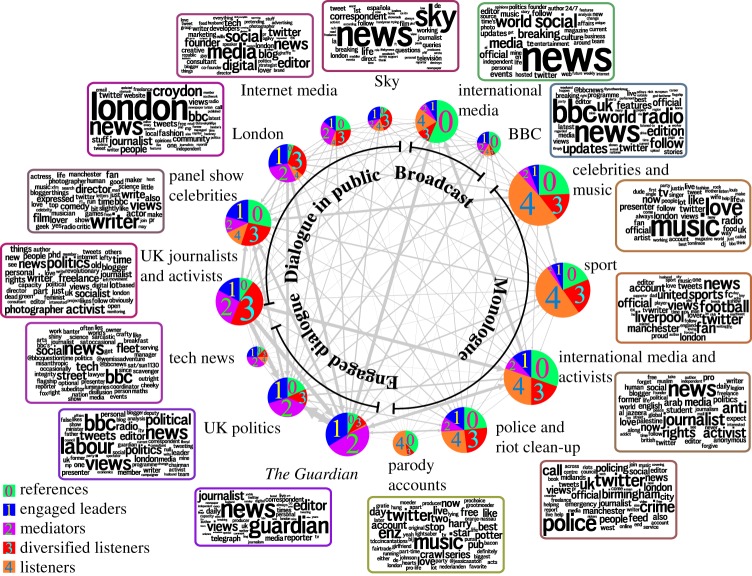
Mix of roles of the 15 interest communities found at *t* = 1.3. The communities reflect a diverse set of topical groupings (see word clouds with the top 50 non-trivial words in the user biographies) and are characterized by different mixes of the five flow roles, as shown by the pie charts. The organigrams range from reference-listener schemes (‘broadcast’ and ‘monologue’) to more balanced dialogue communities (‘engaged dialogue’ and ‘dialogue in public’) in which engaged leaders, mediators and diversified listeners dominate.

Heretofore, our two-pronged flow-based analysis has led to groupings of the nodes according to two criteria: interest communities (at different resolutions) and flow roles. Both perspectives present complementary views of the information in the network and can be combined to characterize the internal organization of interest communities in terms of the mix of roles of their members. Figure 6 presents this integrated view for the 15 interest communities at medium resolution (Markov time *t* = 1.3), and the five node roles found through RBS–RMST. Using a simple *k*-means clustering of their role-mixes, we find that the 15 communities fall into four types of informational organigrams (see the electronic supplementary material). Two of these organigrams broadly conform to communities formed mostly by leaders (‘references’) and their followers (‘listeners’), though with some important differences: ‘monologue communities’ are predominantly composed of references with a set of loyal (non-diversified) listeners with information flowing mostly in one direction (e.g. ‘celebrities/entertainers’, ‘sport’, 'parody accounts’), while in ‘broadcast communities’ most members are references delivering content broadly to a wide variety of users in the network (e.g. ‘BBC’ and ‘international media’). In addition, there exist two organigrams with a more balanced dialogue structure: ‘dialogue in public’, which involves many diversified listeners (e.g. ‘panel show celebrities’, ‘London’, or groups heavily based on Internet interaction such as ‘UK journalists & activists’) and ‘engaged dialogue’, which is dominated by engaged leaders and mediators (e.g. ‘politics’ and ‘*The Guardian*’). These two dialogue organigrams reflect the importance of online interaction in information networks, where bottom-up grassroots associations, bloggers and commentators from the public interact directly with accounts linked to news outlets and official political organizations.

## 3. Discussion

In this work, we have used the Twitter network constructed from the list of influential users during the UK riots of 2011 collected by *The Guardian* to showcase how flow-based methods in directed networks can lead to enhanced insight into the structure of data. Our analysis reveals interest communities into which users fall at different levels of resolution. The interest communities found confirm the relevance of news organizations and media, yet provide a layered view in terms of their focus (UK/international, mainstream/alternative) and of relationships to each other and to the overall network. The enhanced sensitivity of our multi-resolution analysis allows us to uncover small but significant groups related to local organizations or clean-up groups in riot areas which appear close to police and law enforcement groupings. In addition, our analysis reveals groupings that have an unexpected relevance in a network that was selected on the basis of ‘retweeting’ importance during an event of civil unrest. In particular, a significant grouping of celebrities, sports personalities and pop musicians act as the centre of a significant interest community. Also intriguing is the role of interest groups based on humour in such situations, as represented by communities of comedians and parody accounts. Our work points at future studies on how to use this type of analysis to improve and tailor communication strategies during times of unrest, in particular with regards to providing a personalized view of the network from any given vantage point (i.e. from any node or group of nodes) based on the interest distance of information flow. These results can be a starting point to examine textual information and analyse the influence of groups of interest on observed behaviours in this and similar datasets.

Using flow transfer in the network, we find that the Twitter users in this network fall into a palette of five flow roles, whereas interest communities exhibit distinct mixes of such roles reflecting diverse communication patterns within them. Some communities contain one-way communication patterns (e.g. celebrities and their followers), whereas other communities harbour more balanced dialogue patterns. In particular, our analysis highlights the differences between media organizations and their distinct patterns of interaction with the influential users in this network. For instance, international mainstream media tend to fall into the broadcast and monologue categories, as would be expected in a network of UK-based events. On the other hand, the UK and specialized media exhibit a more diverse pattern of interactions with their followers: some of them are highly engaged with mediators and diversified listeners, whereas others largely maintain the more traditional role of publishing content.

This work also highlights the use of multi-scale network analyses, which go beyond local information of individual users towards aggregate global metrics, to deliver an enriched view of information dissemination in social networks, thus uncovering relationships and roles of nodes and providing concise coarse-grained descriptions of the network. We hope that our results (all available in the electronic supplementary material) could be a helpful resource to aid in the study of online interactions during the UK riots of 2011.

More generally, our work highlights the importance of directionality in network analysis. When the notion of flows (e.g. of people, information, energy, goods) is central to a network, ignoring directionality destroys information, ‘blurring’ the structure, especially at the finer levels of resolution, so that key communities (e.g. the BBC, Sky and geographical communities in our analysis) will go undetected. The formulation of community and role detection in terms of flow dynamics thus provides an integrated methodology for the analysis of systems (natural or man-made) with directed network representations.

## Funding statement

M.B.-D., S.N.Y. and M.B. acknowledge support from the UK EPSRC through grant EP/I017267/1 under the Mathematics Underpinning the Digital Economy programme. B.V. was funded by a PhD studentship of the BHF Centre for Research Excellence. M.B.D. also acknowledges support from the James S. McDonnell Foundation Postdoctoral Program in Complexity Science/Complex Systems-Fellowship Award (#220020349-CS/PD Fellow). The authors thank Michael Schaub for many useful conversations.

## Supplementary Material

riots_resub-SI.pdf

## Supplementary Material

RiotsCommunities.xls

## Appendix A. Methods

### A.1. Community detection with directed Markov Stability

We give here a summary of the theoretical ideas and computations underpinning our analysis of interest communities using directed Markov Stability. For a full explanation of the method, see [15,16,20]. The code for the Markov Stability algorithm can be downloaded from http://wwwf.imperial.ac.uk/~mpbara/Partition_Stability/. For an expository article, see [35].

#### A.1.1. Graph-theoretical definitions

Let *A* be the *N* × *N* adjacency matrix of a directed network (*N* = 914 in the riots Twitter network), where *A_i,j_* = 1 if node *i* has an edge to node *j* and 0 otherwise. Note that *A* ≠ *A*^T^ in general. In a directed network, each node has an in-degree (, where  is the *N* × 1 vector of ones) and an out-degree () which are the number of edges directed at the node and departing from the node, respectively.

#### A.1.2. Random walks on directed graphs

A Markov chain on the graph is usually defined by the transition matrix *M* = *D*^−1^*A*, where *D* = diag(*k*_out_) is the diagonal matrix of node out-degrees. For nodes where *k*_out_(i) = 0, the convention is to set *D*(*i, i*) = 1. The evolution of a discrete-time Markov chain is given by

A1

and, alternatively, a Markov process in continuous time is governed by the Kolmogorov equation

A2

Here, **p** denotes the 1 × *N* dimensional probability vector, *I_N_* is the *N* × *N* identity matrix, and *L* is the (combinatorial) Laplacian matrix of the graph. We can view both these processes as defining a random walk taking place on the graph.

To ensure that the random walk is ergodic, we add a ‘teleportation’ component to the dynamics [16] to obtain a new transition matrix

A3

Here  is the probability that a random walker arriving at a node will follow an outgoing edge, while the walker will be ‘teleported’ (i.e. it will jump to any other node in the network chosen at random) with probability (1 − *λ*). In this work, we use *λ* = 0.85 throughout. The probability that any node is visited by a teleported random walker is drawn from a uniform distribution (i.e. each node has the same probability 1/*N* of being visited, though other choices are possible [36]). The *N* × 1 vector *a* is an indicator for dangling nodes: *a*(*i*) = 1 if *k*_out_(*i*) = 0 and *a*(*i*) = 0 otherwise. Upon visiting a dangling node, a random walker will be teleported with probability 1.

In this work, we consider the continuous-time process in equation (A 2) with transition matrix *B*

The steady-state *π* is given by the leading left-eigenvector of *B* (associated by the eigenvalue 1), and the time-dependent transition matrix is .

#### A.1.3. Directed Markov Stability and community detection

We have recently introduced the community detection method known as *Markov Stability*. The basic idea is that the study of the dynamics of diffusion processes on networks can be used to identify meaningful partitions at different resolutions [15,16]. This notion can be illustrated by the example of observing how a droplet of ink would diffuse in a container. If the container has no structure, the ink diffuses isotropically. If the container is compartmentalized, the dye would not spread isotropically but would rather get transiently trapped for longer times in certain parts of the container until it eventually becomes evenly distributed throughout the whole vessel. Hence the time dynamics of this diffusion process provides us with valuable information about the structural organization of the container. A similar idea can be applied to the diffusion on a graph.

From this dynamical perspective, the Markov time acts as a means to scan the structure of the graph *at all scales*, thus providing a dynamical zooming over the structure of the graph. In this process of zooming, the diffusion explores increasingly larger sections of the graph and identifies increasingly coarser partitions. Communities are identified as subgroups within which the probability flow is well mixed yet the flow remains contained over particular time scales. The communities are found by finding the partitions that optimize a time-dependent cost function. As the diffusion progresses, this cost function optimization allows us to rank the goodness of partitions and to identify which partitions are relevant over different time scales. Relevant partitions appear as robust, because they are optimal over extended time intervals and/or in terms of the basin of attraction of the optimization process.

A partition of a network into *C* communities can be encoded into the *N* × *C* indicator matrix *H*, where *H_i,c_* = 1 if node *i* belongs to community *c* and 0 otherwise. Then the Markov Stability of the partition is defined as the trace of the clustered autocovariance of the diffusion process taking place on the graph [15]

A4

where .

We find the communities in the network at all scales by optimizing the Markov Stability (A 4) for any given value of *t* (the Markov time) over all partitions *H*. This is an NP-complete combinatorial problem [15] and to provide optimized solutions, we use the Louvain greedy optimization heuristic [37], which works well in practice. Note that although the original Louvain method is formulated only for symmetric , we have shown that the optimization of the Markov Stability in the case of directed networks can be reformulated in terms of the symmetrized matrix *W* = (*Q* + *Q*^T^)/2, where , which follows from trace (*H*^T^*QH*) = trace (*H*^T^*WH*) [16].

The Markov Stability framework explores the community structure of a network at all scales through the dynamic zooming provided by the duration of the diffusion process *t*: if *t* is small, the diffusion process is short and the optimal partitions consist of many small communities; for larger values of *t*, the diffusion process explores the network further and, consequently, we find fewer and larger communities (figure 1; the electronic supplementary material) [15,16]. The fact that Markov Stability is based on the analysis of flows diffusing in the network allows us to extend seamlessly the analysis of communities to directed networks. In our framework, the defining characteristic of communities is the persistence of flow (contained and well mixed) within the community over a given time scale. Importantly, because Markov Stability is based on the concept of flow, it can detect non-cliquish communities, i.e. communities that are not characterized by density of links but by retention of flow [20]. As we show in the main text and the electronic supplementary material, this property is vital for the analysis of networks with flows of information, particularly in the directed case.

As our method scans dynamically through Markov time, it enables us to find communities defined by their flow patterns at all scales through the optimization of the stability *r*(*H, t*) for a range of *t* spanning several orders of magnitude. Briefly, for each value of *t* we find the partition of the network that maximizes *r*(*H, t*) using the Louvain method [37] from 100 different random initial guesses. The consistency and robustness of the 100 partitions obtained from the optimizations is assessed with the normalized variation of information (VI) [38], as described in [16,20]; see the electronic supplementary material, figure, S2. The VI allows us to gauge the consistency of the partitions obtained from optimizing *r*(*H, t*) at each *t*. A decrease in VI (or an inflection point) at a particular value of *t* suggests relevant community structure at this time scale.

The computational complexity of Markov Stability in its full form (as used here) is slightly better than *O*(*N*^3^) due to the computation of the matrix exponential. This is appropriate for graphs up to several thousands of nodes. For larger graphs, Lambiotte *et al*. [16] and Delvenne *et al*. [35] discuss an approximate (linearized) version of Markov Stability which is approximately *O*(*N*) and can be applied to much larger graphs [39].

### A.2. Finding flow roles in directed networks with role-based similarity–relaxed minimum spanning tree

To classify the nodes according to roles, we combine RBS [17,29] with the RMST algorithm and Markov Stability. We start by creating the RBS matrix, which exploits the directed structure of the graph to obtain a similarity score that measures how alike the flow connectivities of nodes are.

For each node, we obtain a profile vector that contains the number of incoming and outgoing directed paths (incoming and outgoing) of lengths from 1 up to *K*_max_ < *N* − 1 for all nodes. The number of paths corresponding to each node is scaled by a constant and stored as row vectors to create the *N* × 2*K*_max_ matrix:

A5

where , and *λ*_1_ is the largest eigenvalue of *A*. The cosine distances between any two rows of *X*(*α*) (e.g. **x**_*i*_ and **x**_*j*_, corresponding to nodes *i* and *j*) are stored in the *N* × *N* similarity matrix *Y*(*α*):

A6

By construction  with  whenever nodes *i* and *j* have very similar profiles of incoming and outgoing paths of all lengths, i.e. when nodes *i* and *j* play similar roles in the network in terms of flow generation, distribution and consumption. If we choose a small *α*, the terms  converge quickly and the maximum path length (*K*_max_) is small. Hence we would classify nodes according only to their immediate neighbourhoods (in the limit of *α* → 0, nodes are classified according to *k*_in_ and *k*_out_ only). If, on the other hand, *α* is close to 1, the resulting *K*_max_ is larger, thus including global information of the network to classify the nodes. We have followed the iteration prescription detailed in [17] and used *α* = 0.95, which gives *K*_max_ = 133. In its current form, the computational complexity of RBS is slightly better than *O*(*K*_max_*N*^3^). Further algorithmic improvements of this method will be the subject of upcoming publications.

### A.3. The relaxed minimum spanning tree similarity graph

The similarity matrix *Y* is then transformed into a role similarity graph (figure 5) by using the RMST algorithm, which uses a geometric graph embedding based on the iterative addition of relevant edges to the backbone of the minimum spanning tree (MST): edges are only added if there is no alternative path on the tree with a lower distance. This construction attempts to preserve the continuity of the dataset, thus unfolding the structure of the data. The similarity network thus constructed is then analysed for communities using Markov Stability.

In sum, from the original adjacency matrix of the network we use RBS to compute the pairwise similarity between the nodes in the matrix *Y*; then we use the RMST to extract the role similarity graph; and on this graph we perform community detection to obtain the roles of the nodes. We find that the similarity graph of the Twitter network has a robust partition into five types of roles (at Markov time *t* = 97, with zero VI; see the electronic supplementary material). The role classification for every node is provided in the spreadsheet in the electronic supplementary material.

The basic idea of RMST is that weak cosine similarities between high-dimensional vectors are non-informative and do not contribute to our understanding of the structure of the dataset—in high-dimensional space weak similarities are commonplace thus clouding the relationships in the network. Our strategy for the role similarity graph primes the importance of strong similarities: two nodes will not be linked directly in the role similarity graph, if there is already a chain of strong similarities (a weighted path) that links them. More precisely, consider the distance matrix *Z*, where  is the distance between nodes *i* and *j* according to their flow profile vectors. The classical strategy for network construction from a distance matrix is to include an edge between two points if the pairwise distance is less than a threshold value (e.g. if *z_i,j_* < *ɛ*). The problem with this crude strategy is that it does not recover the geometry of the data when the points are not homogeneously distributed [40]. If the threshold is small, then the network will consist of several disconnected components. If the threshold is large, then the network will contain densely connected components, which would take us back to the same problem we had with the full matrix. These problems appear because of the local nature of such an approach, which is exclusively based on local distances.

Instead, we use a global strategy for the construction of the role similarity graph using the RMST algorithm, a method well suited for extracting meaningful networks from datasets that are not homogeneously distributed in a high-dimensional space (in this case ). We begin with an MST as the initial backbone of the graph, and we add edges iteratively using the following simple heuristic (note that the MST is such that the sum of edges in the tree is minimal, and a path in the MST is the path between two nodes that minimizes the maximal edge weight). At each step of the iteration, we consider whether the MST path between any pair of nodes *i* and *j* is a significantly better model than the direct edge *z_i,j_*. If the maximal edge weight in the MST path is significantly smaller than *z_i,j_*, the MST path is considered a better model based on the continuity achieved through short distances. If, on the contrary, the maximal edge weight along the MST path is comparable to *z_i,j_*, then we consider that there is not sufficient evidence to say that the MST path is a better model for data continuity and we add an edge between *i* and *j* in the RMST. Therefore, the edges in the RMST are generated as

A7

where mlink*_ij_* is the maximal edge weight in the MST path between nodes *i* and *j*,  is the distance from node *i* to its *k*th nearest neighbour and *γ* is a positive constant (here, *k* = 1 and *γ* = 0.5). The factor  approximates the local distribution of data points around every point. Our approximation of the local distribution around a point is motivated by the perturbed MST algorithm [41]. This iteration is continued until no more edges are added to the RMST. We call this the *role similarity graph*. The complexity of the RMST algorithm is *O*(*N*^2^).
