# Supplementary material for: Interest communities and flow roles in directed networks: the Twitter network of the UK riots
Source: J R Soc Interface. 2014 Dec 6;11(101):20140940. doi: 10.1098/rsif.2014.0940 (PMC4223916; doi:10.1098/rsif.2014.0940)
Supplement: riots_resub-SI.pdf [file rsif20140940supp1.pdf]

Interest communities and flow roles in directed networks: the Twitter network of the UK riots

Mariano Beguerisse-Díaz [1,2]*, Guillermo Garduño-Hernández [3], Borislav Vangelov [1],
Sophia N. Yaliraki [2], Mauricio Barahona [1,†]
[1]Department of Mathematics, Imperial College London, London SW7 2AZ, United Kingdom.
[2]Department of Chemistry, Imperial College London, London SW7 2AZ, United Kingdom.
[3]Sinnia, Mexico City, Mexico.

Supplementary Information

1 The UK riots Twitter network

1.1 The Guardian's list of influential user during the UK riots of 2011

Recently, the British newspaper The Guardian announced that it possessed a database of 2.5 million Twitter messages (tweets) related to the riots that took place in England during the summer of 2011. In December 2011, The Guardian made public a list of the 1000 most influential Twitter users according to the number of forwarded (re-tweeted) status updates during the riots [1]. This diverse list of Twitter users includes large news and media outlets, social and political organisations, as well as personal accounts of politicians, journalists, activists, celebrities, and parody accounts. In addition, a considerable fraction of the list is formed by regular individuals whose tweets received attention during the riots, and about whom little is known. The list provided by The Guardian contains the Twitter user name, the number of re-tweets, and a mini-biography of at most 140 characters which Twitter users can provide to describe themselves.

1.2 Creating the Twitter network from The Guardian's list

We created the network from The Guardian's list (Figure 1 of the Main Text) by mining Twitter in February 2012 and obtaining the "friends" of all users in the list, i.e., the Twitter accounts to which each user is subscribed, which is publicly available information. A directed, unweighted network was obtained by intersecting the list of friends with the members of the list. In this network, the information flows from target to source along each edge, i.e., opposite to the direction of the edge, which represents the declared interest of the source node (Fig. S1A). The resulting directed network has a giant component of 914 nodes.

*m.beguerisse@imperial.ac.uk
†m.barahona@imperial.ac.uk

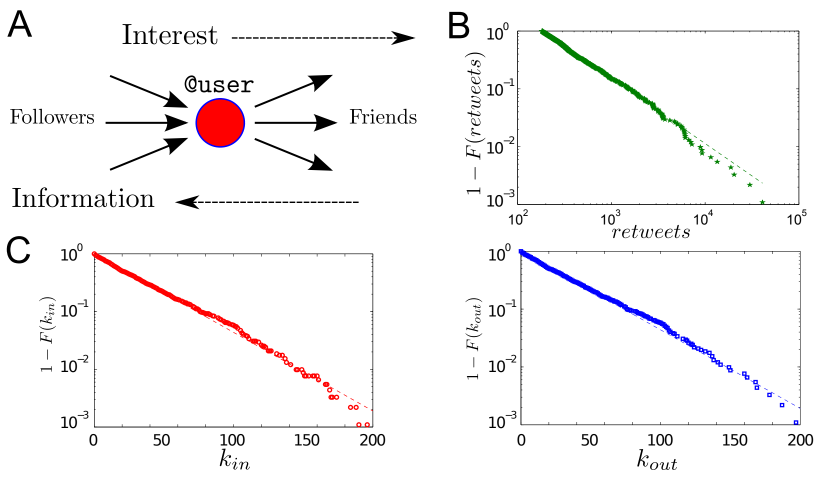

Figure S1: Some properties of the UK riots' influential Twitter users network. **A**: A directed network is created by intersecting each user's friends with the user list. The interest (or attention) is directed at friends, and information travels in the opposite direction (from the friend to the follower). **B**: Cumulative re-tweet distribution. The dotted line is the fit $retweets^{-\alpha}$, where $\alpha = 2.12$. **C**: Cumulative in-degree (left, in red) and out-degree (right, in blue) distributions. The dashed lines show fits of the data to $e^{-\lambda_{in}k_{in}}$ and $e^{-\lambda_{out}k_{out}}$, with $\lambda_{in} = 0.0313$ and $\lambda_{out} = 0.0312$.

The remaining 86 nodes are either completely disconnected (i.e., they do not follow nor are followed by anyone on the list), or their accounts have since been discontinued. All the subsequent work in this paper uses this 914-node network.

1.3 Some statistics of the network

The distribution of the number of retweets of the members of the list (Fig. S1B) is compatible with a power-law with exponent $\alpha \sim 2.12$ ($p = 0.75$, using the criterion in Ref. [2]), which is consistent with previous analyses of Twitter data sets [3]. The cumulative in- and out-degree distributions of the connected component are shown in Fig. S1C. Although both distributions appear exponential with, respectively, parameters $\lambda_{in} \approx 0.0313$ and $\lambda_{out} \approx 0.0312$, a Kolmogorov-Smirnov statistic does not provide statistically significant support for this hypothesis ($p < 0.1$). The distributions of k_{in} and k_{out} in this data are less skewed than other published results [4]. Note also that the in- and out-degrees are similarly fat-tailed, in contrast with other studies in which k_{in} was found to be more skewed than k_{out} [5]. These differences of our distributions with other published reports may be due to the fact that our relatively small user list corresponds to a subset of users with a high number of retweets in The Guardian's riot tweet database, and may not be representative of the wider network of all Twitter users.

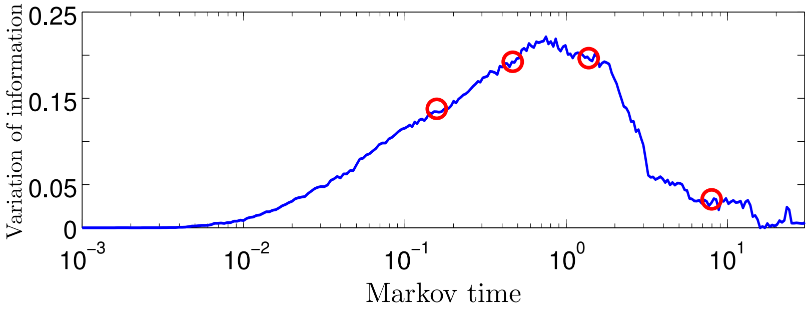

Figure S2: Variation of information of 100 Louvain optimisations of directed Markov Stability at each Markov time. The red circles indicate the Markov times of the communities presented in the main text.

2 Interest communities at different levels of resolution in the Twitter network of the 2011 UK riots

As a relevant application of current interest, we use the Markov Stability framework to analyse a directed graph derived from a social network: the Twitter network derived from The Guardian list of influential users during the UK riots.

In Fig. 1A of the Main Text we show the number of communities and in Fig. S2, we show the variation of information (VI) obtained after optimising stability for Markov times between 10^{-3} and $10^{1.5}$. Below we provide some examples of communities found at different Markov times (marked with red circles on the variation of information (VI) in Fig. S2). These examples were highlighted due to the relative robustness of the communities, as well as a means to showcase the different types of communities found at different levels of resolution.

In the Supplementary Spreadsheet, we provide a spreadsheet with all the communities found at all Markov times. The convention we have followed to name the communities is T[Markov time]-C[community number].

2.1 Communities at high level of resolution

At short Markov times ($t = 0.15$), we find a revealing partition with 149 communities. Though granular, the community structure of the partition shows interesting features. Some of the communities in this partition correspond to a precise geographical location in England or within a city (Fig. S3). For example, there are communities from Hackney (where the riots began) and Croydon. The latter includes the account of London's Mayor (mayoroflondon). Other geographically homogeneous communities are from the Midlands, Liverpool, and Manchester (T0.15-C10, T0.15-C11 and T0.15-C28).

Coexisting with those geographical communities at this level of resolution, we also find journalists and media outlets in other communities defined by their affiliation. Figure S4 shows communities from The Daily Telegraph, The Independent, ITV, Sky and the BBC. Other interesting communities in this partition are, for example, formed by UK activists (T0.15-C0), and another formed by the Anonymous Internet activist group (T0.15-C12), see the Supplemental Material.

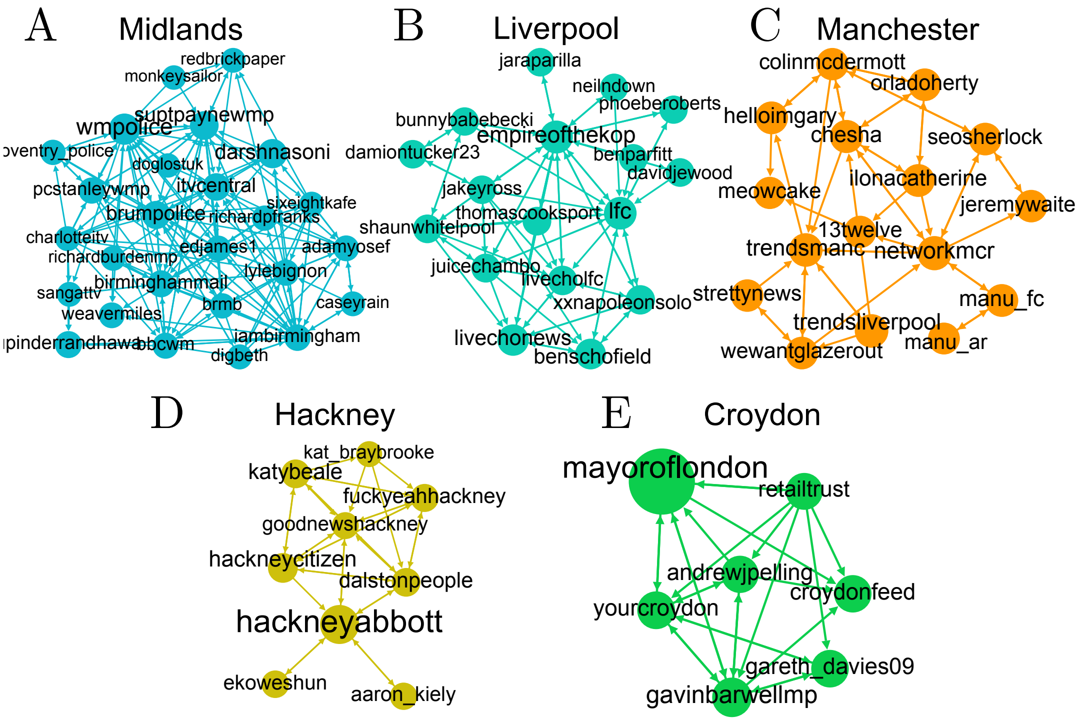

Figure S3: Geographical communities found at high level of resolution ($t = 0.15$). Communities where most members identify themselves as from the Midlands (A), Liverpool (B), Manchester (C), Hackney (D), and Croydon (E).

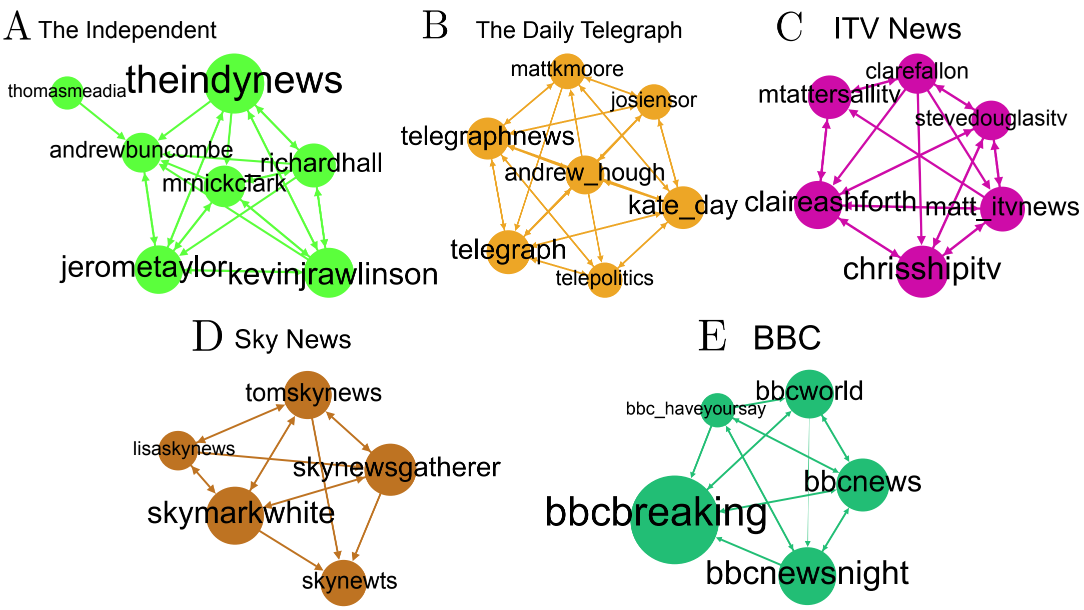

Figure S4: Media communities found at high level of resolution ($t = 0.15$): The Independent (A), The Daily Telegraph (B), ITV (C), Sky News (D), and BBC (E).

2.2 Communities at medium level of resolution

As the Markov time grows, the communities become coarser. In Fig. S5, we show the partition of the network at Markov time $t = 0.5$, when there are 48 communities in the riot network. The largest community in this partition (T0.5-C0) with 57 members is the 'Sports' community. Other examples of communities found at this level of resolution include a community of 'Comedians, writers and presenters'; a 'Parody' community; a community of 'Music journalists and artists'; a community of 'Police forces and crime journalists'; a 'London' community; a community of 'Activist, students and journalists with a focus on the Middle East'; and an 'Online media' community of (mostly) Internet media outlets, individuals and companies.

2.3 Communities at a coarser level of resolution

When the Markov time is longer ($t = 1.3$) we find a partition into 15 communities in the network. In Fig. 6 of the Main Text we present a coarse-grained overview of the communities, their relationships, and their word cloud self-descriptions. This partition, its communities, and the global view of the network it provides are discussed at length in the Main Text.

2.4 Communities at low level of resolution

Finally, at long Markov times ($t = 7.4$), we find the partition into 4 communities shown in Fig. 1C of the Main Text. At this level of resolution there are three large communities consisting of nodes with prevailing

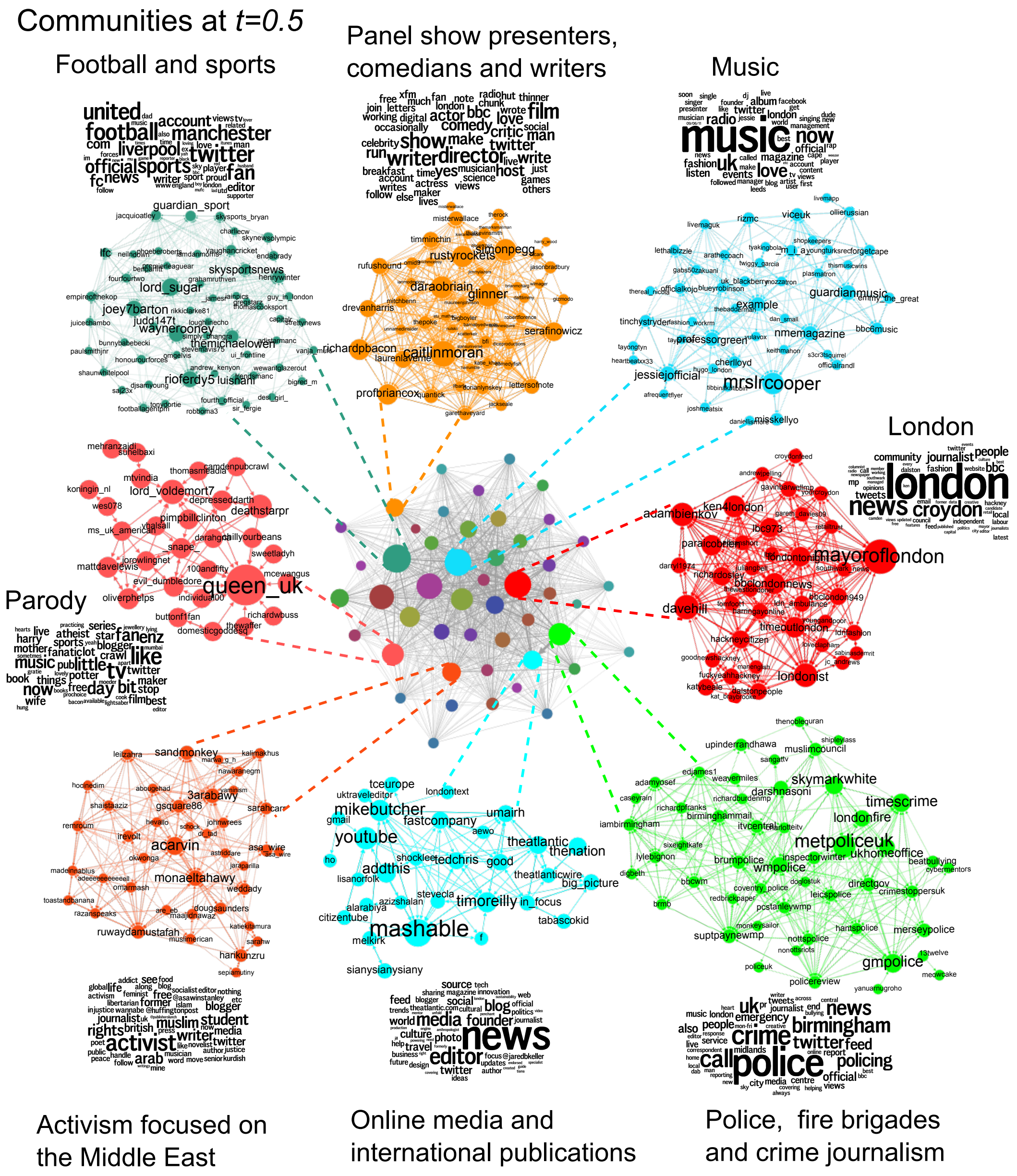

Figure S5: Showcase of communities found at medium resolution ($t = 0.5$). At this Markov time there are 48 communities including one of footballers and sports-related accounts (T0.51-C0), panel show presenters and comedians (T0.51-C1), music (T0.51-C4), parody accounts (T0.51-C11), London (T0.51-C7), activism focused on the Middle East (T0.51-C5), online media (T0.51-C10), and police forces, fire brigades and crime journalism (T0.51-C3).

interest in ‘Media an entertainment’ (T7.4-C0), ‘UK politics, activism and journalism’ (T7.4-C1), and ‘International media and activism’ (T7.4-C2). In stark contrast, the fourth community (T7.4-C3) is small and contains only 26 nodes mostly related to the BBC.

2.5 The change in the community structure when directionality is ignored

As discussed in the Main text, the community structure detected is significantly different if the directionality of the edges is ignored. This phenomenon was exemplified through two examples: the BBC community (in Fig. 2 of the Main Text, which was heavily affected when directionality was neglected) and the Monbiot community (in Fig. 3 of the Main Text, which remained relatively unaffected).

To complement this view, we show here the comparison of the communities found at *all* Markov times for the directed riots network and two undirected versions of it: an undirected network obtained by simply ignoring the direction of the edges, and a *symmetrised* version of the network whose adjacency matrix is $A + A^T$ (i.e., reciprocal edges have twice the weight of nonreciprocal ones). In Fig. S6A-B, we show that the partitions found in the directed network are different from those found in the undirected and symmetrised versions across all Markov times, whereas both symmetrised versions are similar to each other (Fig. S6C). The differences between the directed and undirected versions are high at small Markov times and, as expected, they become smaller as the Markov time grows (i.e., at lower resolutions). Hence the most prominent effect of ignoring directionality in this network is to blur the fine structure of the flow communities.

For ease of comparison, the full sets of partitions for both the directed and undirected graphs at all Markov times is given in the Supplementary Spreadsheet.

2.6 Comparison with other community detection methods—Infomap

As a comparison with our directed Markov Stability methodology, we analysed the community structure of the directed Twitter network using Infomap [7, 8], as downloaded from `http://www.mapequation.org/`. Infomap is a well-known method for community detection in directed and undirected networks based on information compression, which has been shown to perform well in some benchmarks with clique-like communities.

In this case, Infomap obtains partitions only at two levels of resolution. The finer of these two partitions consists of 342 communities: 318 communities contain only one node; 50 communities contain only two nodes; and the largest community contains 60 nodes. The coarser of these two partitions has 60 communities: 26 communities still have only one node while the largest community has 342 nodes, i.e., more than a third of the nodes in the network. Hence, in this case, the communities obtained by Infomap lead to an over-partitioned description of our Twitter network.

We provide all the partitions obtained with Infomap in the Supplemental Spreadsheet.

These results of Infomap are consistent with the analysis presented in Refs. [9, 10], where it is shown that Infomap is a one-step method, which is highly efficient for the detection of clique-like communities but which may lead to over-partitioning when the communities are non-clique like. In contrast, Markov Stability makes use of the full transients (i.e. the complete dynamics with paths of all lengths, as shown in Fig. S8A and Fig. 1 from the Main text) to unfold the community structure across scales. This tendency of Infomap to over-partitioning in some networks is signalled by a large compression gap [9] with respect

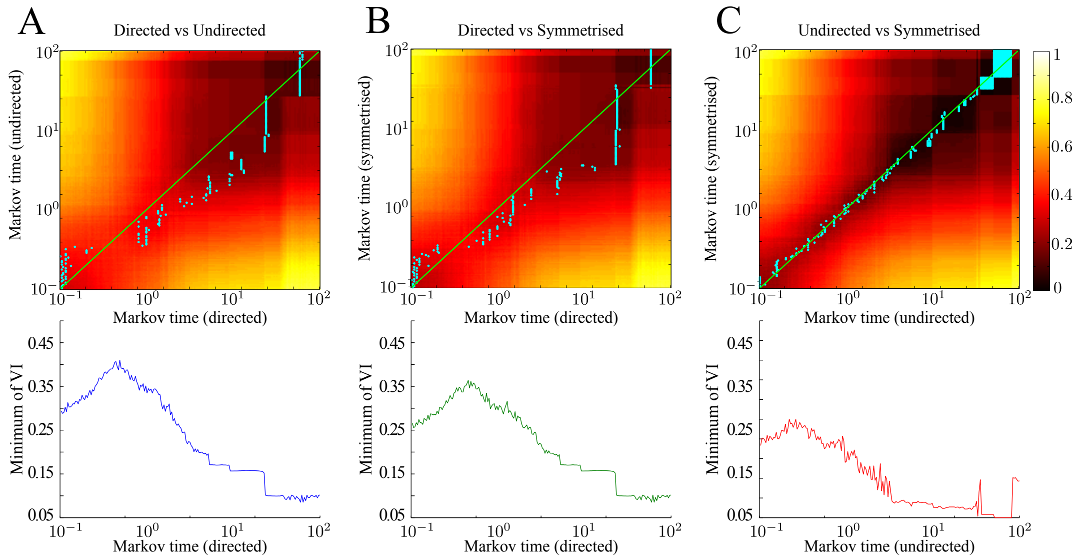

Figure S6: The directed and undirected versions of the Twitter network show distinct community structures across Markov times. **A**: Comparison between the directed version of the network and the undirected one. Top: Variation of information (VI) between the partitions at all Markov times. Low values of VI indicate high similarity. Each row contains the VI between the undirected partition to all directed partitions. The minimum of each row is shown in light blue. Bottom: These minimum values of the VI from each row in the top plot are shown as a function of Markov time. The relative high values of the VI indicate that the directed partitions are different from the undirected ones across Markov times, but less so at large Markov times. **B**: The comparison between the directed network and another undirected version of the same network (the $A+A^T$ symmetrised network) shows similar features. **C**: The comparison between these two *undirected* versions of the network (i.e, the standard undirected and symmetrised networks) show that the partitions are highly similar at all Markov times, as can be seen by the low values of the VI between the closest partitions, and their alignment along the diagonal of the VI plot (top).

to the optimal compression. In this particular case, the code-length of the Infomap partition is 8.3977 bits (after 500 trials) and the compression gap is 0.3647, which is more than three times larger than is achieved for clique-like communities [9]. Hence, although in other instances and benchmarks Infomap performs very well, for this network it does not produce the nuanced description at different levels of resolution that our method delivers through the full use of flow transients [9, 10]. If the direction of the edges is ignored, this over-partitioning effect of Infomap is even more striking. For the undirected graph, Infomap obtains two partitions (code-length: 9.22 bits, compression-gap: 0.455), with 800 communities in the finer partition and 6 communities in the coarser partition, one of which contains 894 nodes.

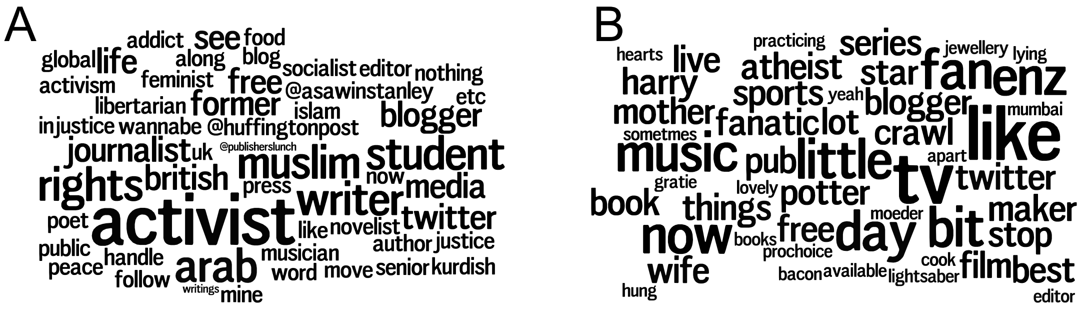

Figure S7: Example word clouds created from the 50 most frequently-used words in two communities from Fig. S5. **A**: Word cloud from the biographies of an activist community (T0.5-C5). More frequent words appear larger than less-frequent ones (function words ignored). **B**: Word cloud of the Parody account community (T0.5-C11) whose members are linked by their interests but do not use a common vocabulary to describe themselves collectively. In this case the word frequencies do not help establish the nature of the community.

3 Self-descriptions from Twitter biographies

Interpreting the communities found in the analysis by looking through all their members is mostly impractical. As an aid to assess the quality and intrinsic content of the communities found, we tap into the information contained in the mini-biographies provided by the users. Our premise is that we can learn valuable information about a community from the small texts that the members write about themselves. To do this in a more systematic manner, we collect all the biographies of a community in a single file (removing urls, emails, numbers, function words, and other nonstandard characters) and count the occurrences of all words. We then compile the most frequently used words as an aid in the characterisation of each community and construct word cloud visualisations [6] of the word-frequencies of the 50 most used words in each community. The word frequencies (and their word-cloud representation) acts as a 'self description' of each community.

In some cases, the self-descriptions of the users in a community share highly indicative words, which appear prominently in the word clouds. Figure S5 shows that the word clouds of several communities at $t = 0.5$ all represent well their character. For example, the members of the 'Middle-East activism' community describe themselves using a consistent vocabulary representing their interests, as shown in Fig. S7A.

On the other hand, other communities are more heterogeneous in the self-descriptions of their members. For instance, the members of the 'Parody' community do not use a common vocabulary to describe themselves (Fig. S7B), so in this case the word frequencies do not help establish the nature of the community. Indeed, this group does not share a common thematic content but are otherwise linked by their acting as ironic reflections of a variety of celebrities. This is reflected in their identification as an interest community. If we analyse the membership of the community carefully, one can see that the community contains many parody accounts (e.g., parodies of the Queens of England and the Netherlands, and Star Wars and Lord of the Rings characters).

In general, given the small amount of text available in the self-descriptions, the word frequencies and word-clouds must be used judiciously. However, they can be of great aid in providing a simple visual interpretation of the communities, as shown in the figures in this Supplemental Information and the Main Text.

4 Using the RMST-RBS similarity graph to uncover roles in the network

The RMST-RBS graph thus constructed is a new graph (undirected and unweighted), which captures geometrically how similar two nodes are, based on their vectors of incoming and outgoing flow profiles. Clearly, this role similarity graph is distinct from the original graph that originated it: two nodes are connected in the role similarity graph only if they have similar profiles of incoming and outgoing paths in the Twitter network, *regardless of whether they are neighbours in the original network.* Figure 5 in the Main Text shows the role similarity graph constructed from the Twitter riot network.

We apply a graph-theoretical community detection method (in this case undirected Markov Stability) to the role similarity graph to find if there any significant groups of nodes with similar flow profiles, without imposing their number or type a priori. Figure S8A shows that the Markov Stability analysis of the role similarity graph finds a very robust partition into five communities of approximately similar sizes at $t = 97.712$. At this Markov time, the variation of information is 0, which means that in *all of* the 100 times we ran the community detection algorithm we obtained the exact same partition. These five communities in the role similarity graph correspond to classes (or types) of roles in the network. In the Supplemental Material we provide the full classification of nodes according to these roles.

To interpret the five clusters found by our analysis, we examine *a posteriori* different characteristics of their members. Figure S8B shows the mean in and out degree in the original Twitter network of the nodes in each class found. Two of the groups found have higher mean in-degree (i.e., Twitter followers) than out-degree, while for the other three groups the reverse is true (i.e., they follow more than they are followed by). If we coarse-grain the original Twitter network lumping together all the nodes with the same role (as in Fig. 5 of the Main Text), a striking pattern of connectivity is revealed with classes of nodes mostly receiving attention (sinks of interest or sources of information), other classes mostly behaving as sources of interest (recipients of information) and other classes in between. This leads to our renaming our role classes as: references, engaged leaders, mediators, diversified listeners, and listeners (see Main Text for more details). Finally, the PageRank distributions shown in Fig. S8C also help illustrate the differences between leader, follower, and mediator roles, but would not be able to discriminate between the roles obtained from our analysis of the role similarity graph.

4.1 Comparison to other classic notions of roles in social network analysis

The notion of node roles in graphs has been studied from different perspectives, especially in social network analysis. A classic example is *structural equivalence* (SE) in which two nodes have the same role if they share the same neighbours [11, 12], i.e., if they are swapped the network remains the same. One can compute how similar (in the SE sense) are two nodes based on the number of common neighbours. SE roles are thus based on computing the number of common immediate neighbours and bear no resemblance

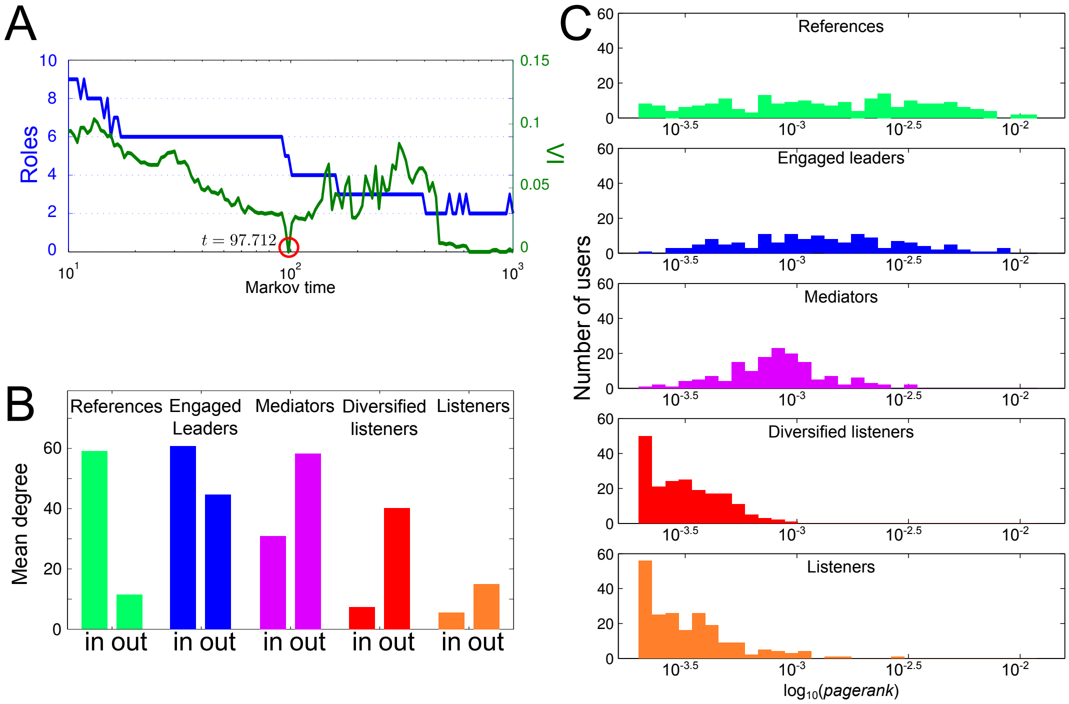

Figure S8: **A**: Community detection of the role similarity graph using Markov Stability. The blue line shows number of communities and the green line shows the variation of information. A robust partition into five communities is detected at Markov time $t = 97.712$ corresponding to the five roles in the Twitter network. **B**: The in- and out-degrees of the five clusters found reflect their characterisations as: references, engaged leaders, mediators, diversified listeners, and listeners. **C**: Histogram of PageRank values in each of the five role classes. The listener categories contain mostly nodes with low page rank while the high PageRank values are concentrated in the reference and engaged leader categories.

to the flow roles detected via the RMST-RBS approach. In particular, our approach allows nodes with no common neighbours to have the same role, counter to the SE definition.

Another classic notion of role in social networks stems from the theory of *regular equivalence* (RE). RE uses node colorations (or labellings) to find groups of nodes with the same role [13, 14, 15]. Suppose u is a node in the network with in-neighbours $N_i(u) = \{j : A_{j,u} = 1\}$ and out-neighbours $N_o(u) = \{j : A_{u,j} = 1\}$. The colour of u is $C(u)$ and the colour of the in and out-neighbourhoods are $C(N_i(u))$ and $C(N_o(u))$ respectively. A coloration is said to be (exactly) regular [14] if for any two nodes u and v

$$C(u) = C(v) \Rightarrow \begin{cases} C(N_i(u)) = C(N_i(v)) & \text{and} \\ C(N_o(u)) = C(N_o(v)). \end{cases} \tag{1}$$

In a regular (or approximately regular) coloration of a network, nodes with the same colour are said to have the same role [15, 16]. In its strict sense, the RE definition of role is combinatorial (and thus lacking robustness in many real-world networks networks). Furthermore, it is only based on the consideration of the coloration of immediate neighbourhoods of each node. Hence it leads to a very different classification of roles to that obtained with the RMST-RBS algorithm.

We have obtained the roles of nodes in the riots network obtained using RE models, and compared them to the RMST-RBS roles obtained above. To obtain the RE classes we use two well-known algorithms: REGE [17], which obtains similarities between the nodes based on RE, and EXCATRE [15] which produces a sequence of regular (or approximately regular) colorations. We have included the EXCATRE and REGE partitions in the Supplemental Spreadsheet.

The EXCATRE algorithm applied to the riots network finds only one non-trivial coloration with 734 roles. (Two trivial colorations are also obtained: all nodes with the same colour and each node with its own colour.) On closer investigation, the roles identified by EXCATRE correspond trivially to nodes with identical in and out-degree, and hence it corresponds to considering only immediate neighbourhoods in the graph (i.e., it would be found by RMST-RBS setting both α and K_{max} equal to 1 so that only paths of length one are considered). In our analysis above, we used $\alpha = 0.95$ (which converges at $K_{max} = 133$), and the roles obtained by RMST-RBS incorporate global flow information from the graph.

The REGE algorithm iteratively constructs a similarity matrix between nodes based on the similarities between their neighbours. The similarity matrix is then clustered using hierarchical clustering techniques to obtain roles [16]. We apply the REGE algorithm to the riots network (which converges after 6 iterations) and obtain five clusters (or roles) with 806, 59, 46, 2 and 1 nodes each. These roles are not informative: the cluster with 59 nodes contains all the source nodes (in-degree 0); the cluster with 46 nodes contains all the sink nodes (out-degree 0); while almost all the nodes in the network fall in the cluster with 806 nodes which lies between them. The two small clusters with 2 and 1 nodes lie in slightly different positions with respect to the sink and node clusters. As with EXCATRE, these roles are the result of an analysis based on immediate neighbourhoods (in and out-paths of length one).

Although RE-based methods and RMST-RBS attempt to find roles in networks, their conceptual foundation is different. Both approaches aim to identify the 'types' of nodes that exist in the network (allowing for the possibility that two nodes on opposite ends of the network and no common neighbours could be of the same type) but they use different information to do so. RE-based methods analyse the similarities between neighbourhoods under colorations, while RMST-RBS identifies similarities in the transient pattern of long-range flows though each node. Because of the use of flows at all scales in the network, from local to

global neighbourhoods, the RMST-RBS method provides a more balanced classification into node classes. In addition, RMST-RBS is less sensitive to small changes in connectivity, and thus more robust for the analysis of realistic networks. For example, if we create an edge from a node in the 806-node REGE cluster to the 59-node cluster of sources (e.g., when a Twitter user decides to 'follow" another user), this node would change roles immediately. Hence in this specific context, a RE-based analysis of the network is non robust.

5 Integrating interest communities and roles: informational organigrams

Our two analyses (interest communities and user roles) can be brought together to classify the informational organisation of communities, as given by the different mix of roles within each community. In Fig. 6 of the Main Text we show that the 15 communities obtained at $t = 1.3$ can be broadly classified into four organigrams, which go from a purely broadcast community (of references and listeners) to other communities that involve dialogue between engaged leaders, mediators and diversified listeners.

The four organigrams were found by using the mix of roles of each community (a five-dimensional vector containing the proportion of nodes in the community that belong to each role-class) and performing a simple k-means clustering on the communities. In other words, each community can be represented by a point inside the unit cube in $\mathbb{R}^5$, and with k-means we identify the clusters of communities whose role mixes are similar.

References

[1] Evans L, Vis F. 200 most influential Twitter users during the riots: are you on the list?; 2011. Available from: `http://www.guardian.co.uk/news/datablog/2011/dec/08/riot-twitter-top-200`.

[2] Clauset A, Shalizi C, Newman M. Power-Law Distributions in Empirical Data. SIAM Review. 2009;51(4):661–703.

[3] Zhou Z, Bandari R, Kong J, Qian H, Roychowdhury V. Information resonance on Twitter: watching Iran. In: Proceedings of the First Workshop on Social Media Analytics. SOMA '10. New York, NY, USA: ACM; 2010. p. 123–131.

[4] Kwak H, Lee C, Park H, Moon S. What is Twitter, a social network or a news media? In: Proceedings of the 19th international conference on World wide web. WWW '10. New York, NY, USA: ACM; 2010. p. 591–600.

[5] Wu S, Hofman JM, Mason WA, Watts DJ. Who says what to whom on twitter. In: Proceedings of the 20th international conference on World wide web. WWW '11. New York, NY, USA: ACM; 2011. p. 705–714.

[6] Wordle. Available from: `http://www.wordle.net`.

[7] Rosvall M, Bergstrom CT. Maps of random walks on complex networks reveal community structure. Proc Natl Acad Sci U S A. 2008 Jan;105(4):1118–1123.

[8] Rosvall M, Bergstrom CT. Multilevel compression of random walks on networks reveals hierarchical organization in large integrated systems. PLoS One. 2011;6(4):e18209.

[9] Schaub MT, Lambiotte R, Barahona M. Encoding dynamics for multiscale community detection: Markov time sweeping for the map equation. Phys Rev E Stat Nonlin Soft Matter Phys. 2012 Aug;86(2 Pt 2):026112.

[10] Schaub MT, Delvenne JC, Yaliraki SN, Barahona M. Markov Dynamics as a Zooming Lens for Multiscale Community Detection: Non Clique-Like Communities and the Field-of-View Limit. PLoS ONE. 2012 02;7(2):e32210.

[11] Lorrain F, White HC. Structural equivalence of individuals in social networks. The Journal of Mathematical Sociology. 1971;1(1):49–80.

[12] Leicht EA, Holme P, Newman MEJ. Vertex similarity in networks. Phys Rev E. 2006 Feb;73:026120.

[13] White DR, Reitz KP. Graph and semigroup homomorphisms on networks of relations. Social Networks. 1983;5(2):193 – 234.

[14] Everett MG, Borgatti SP. Regular equivalence: General theory. The Journal of Mathematical Sociology. 1994;19(1):29–52.

[15] Everett MG, Borgatti SP. Exact colorations of graphs and digraphs. Social Networks. 1996;18(4):319 – 331.

[16] Luczkovich JJ, Borgatti SP, Johnson JC, Everett MG. Defining and Measuring Trophic Role Similarity in Food Webs Using Regular Equivalence. Journal of Theoretical Biology. 2003;220(3):303 – 321.

[17] Borgatti SP, Everett MG. Two algorithms for computing regular equivalence. Social Networks. 1993;15(4):361 – 376.
